# Identification and epidemiology of a novel *Hepacivirus* in domestic ducks in Hunan province, China

**DOI:** 10.3389/fvets.2024.1389264

**Published:** 2024-05-02

**Authors:** Jin-Tao Chen, Kang-Jing Chen, Kang-Wei Wu, Shan-Hong Yi, Jian-Wei Shao

**Affiliations:** ^1^School of Basic Medical Sciences, Wuhan University, Wuhan, China; ^2^School of Medical Technology, Shangqiu Medical College, Shangqiu, China; ^3^Department of Microbial Testing, Hengyang Center for Disease Control & Prevention, Hengyang, China; ^4^School of Life Science and Engineering, Foshan University, Foshan, China

**Keywords:** *Hepacivirus*, duck, genetic diversity, epidemiology, China

## Abstract

The genus *Hepacivirus* comprises a diverse range of genetically distinct viruses that infect both mammalian and non-mammalian hosts, with some posing significant risks to human and animal health. Members of the genus *Hepacivirus* are typically classified into fourteen species (*Hepacivirus A–N*), with ongoing discoveries of novel hepaciviruses like *Hepacivirus P* and *Hepacivirus Q*. In this study, a novel *Hepacivirus* was identified in duck liver samples collected from live poultry markets in Hunan province, China, using unbiased high-throughput sequencing and meta-transcriptomic analysis. Through sequence comparison and phylogenetic analysis, it was determined that this newly discovered *Hepacivirus* belongs to a new subspecies of *Hepacivirus Q*. Moreover, molecular screening revealed the widespread circulation of this novel virus among duck populations in various regions of Hunan province, with an overall prevalence of 13.3%. These findings significantly enhence our understanding of the genetic diversity and evolution of hepaciviruses, emphasizing the presence of genetically diverse hepaciviruses duck populations in China. Given the broad geographical distribution and relatively high positive rate, further investigations are essential to explore any potential associations between *Hepacivirus Q* and duck-related diseases.

## 1 Introduction

The genus of *Hepacivirus*, along with the genera *Flavivirus*, *Pegivirus* and *Pestivirus*, is currently classified within the family *Flaviviridae*, which includes a genetically diverse group of human and animal pathogens (1, 2). Members of the genus *Hepacivirus* are enveloped viruses with unsegmented, single-stranded, positive-sense RNA genomes that are approximately 10 kb in length (1). These genomes contain the 5′ untranslated regions (UTR) and 3′ UTR, as well as a single large open reading frame (ORF) that encodes a polyprotein (3). The polyprotein is cleaved by signal peptidase, NS2/NS3 protease, and NS3 protease enzymes, resulting in the production of three structural proteins (Core, E1, and E2) and seven nonstructural proteins (p7, NS2, NS3, NS4A, NS4B, NS5A, and NS5B) (4).

*Hepacivirus* was initially identified in 1989 as a human pathogen, with humans believed to be its sole natural host for a long time (5). However, since 2011, novel and genetically diverse hepaciviruses have been found in a wide range of mammalian and non-mammalian hosts (6). Moreover, recently discovered hepaciviruses named *Hepacivirus P* and *Hepacivirus Q* were detected in long-tailed ground squirrels and domestic ducks in China, respectively (7–9). These distinct hepaciviruses found in different host species are currently mainly classified as *Hepacivirus A*–*N* based on their phylogenetic relationships and host range (2).

The first identification of *Hepacivirus* in avian species was documented in 2019, specifically in domestic ducks across various areas in China (9). This virus has been associated with significant declines in egg production, with viral RNA detection rates ranging from 38.5 to 88% in different areas (9). Subsequently, additional novel hepaciviruses were also discovered in avian species (10, 11). More recently, another novel *Hepacivirus*, named *Hepacivirus Q*, was identified in domestic ducks in China (8), highlighting the genetic diversity of hepaciviruses in avian species. In this study, a new subspecies of *Hepacivirus Q* was discovered in domestic ducks from Hunan province, China using unbiased high-throughput sequencing and meta-transcriptomic analysis. Furthermore, the prevalence of this novel virus in duck populations in specific regions of Hunan province, China was examined. This study not only enhances our understanding of the genetic diversity and evolution of hepaciviruses but also underscores the importance of investigating whether *Hepacivirus Q* is associated with any duck disease.

## 2 Materials and methods

### 2.1 Sample collection and meta-transcriptome sequencing

**Figure 1:**
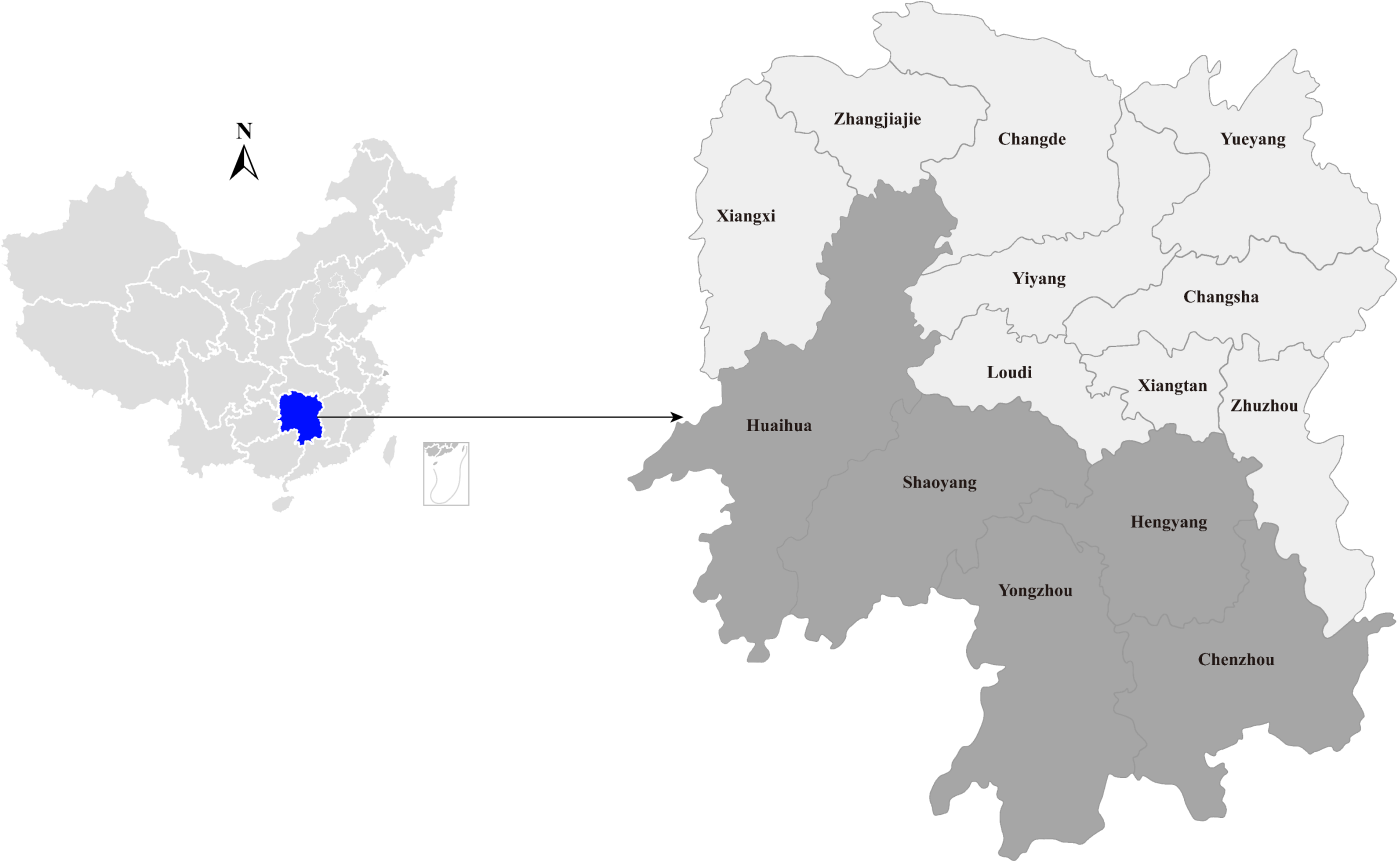
Geographic maps showing the location of sampling sites where the duck liver samples collected in this study. This map was plotted by combination of Surfer software version-4 (Golden Software, United States) and Adobe illustrator version CC2017 (Adobe, United States).

In a poultry viral agent discovery project, 40 duck liver samples were collected from live poultry market located in Hengyang city, Hunan province, China, between August and September 2023 (Figure 1). Approximately 50 mg of liver tissue was homogenized with 500 μL sterile phosphate-buffered saline (PBS). Total RNA was extracted from 200 μL homogenates by using RNAiso Plus reagent (TaKaRa, Dalian, China) and subsequently purified using the RNeasy Plus Mini Kit (Qiagen, Germany). The quantity and quality of the extracted RNA were assessed using a NanoDrop 2000 (Thermo Fisher Scientific, Waltham, United States). Subsequently, the individual RNA solutions were pooled in equal quantities, and the quality of the pooled RNA was evaluated using an Agilent 2,100 Bioanalyzer (Agilent Technologies) before library construction and sequencing.

The RNA library preparation followed the methodology as previously described (12, 13). Briefly, ribosomal RNA (rRNA) was depleted using a Ribo-Zero-Gold (Epidemiology) kit (Illumina Inc., United States) following the manufacturer’s instructions. The remaining RNA was fragmented, reverse-transcribed, adapted, and purified using the TruSeq total RNA library preparation kit (Illumina Inc., United States). The quality of the library quality was assessed using the Qubit high-sensitivity RNA/DNA assays (Thermo Fisher Scientific) and the Agilent 2,100 Bioanalyzer (Agilent Technologies). Paired-end sequencing with 150-bp read length was conducted on the Illumina Hiseq2500 platform. All library preparation and sequencing procedures were carried out by Novogene (Tianjin, China).

### 2.2 Bioinformatics analyzes and viral genome sequence determination

The sequencing reads were demultiplexed, trimmed for the adaptor, and subjected to quality control using the fastp program (14). Subsequently, *de novo* assembly was performed using the Megahit program (15) with default parameters. The resulting contigs were compared against the entire non-redundant protein (nr) database downloaded from GenBank using the diamond blastx program (16) with an e-value threshold of 1e–3. Viral contigs with unassembled overlaps or originating from the same scaffold were merged using the SeqMan program implemented in the Lasergene software package (version 7.1, DNAstar).

To confirm the assembly results, reads were mapped back to the target contigs with Bowtie2 (17), and assembly errors were inspected using Integrated Genomics Viewer (IGV). Any gaps between these contigs were filled by reverse transcription PCR (RT-PCR) and Sanger sequencing (Table 1). The virus genome termini were determined using 5′/3′ RACE kits (TaKaRa, Dalian, China) following the producer’s instructions. The final virus genome sequence was obtained by consensus mapping assembly and confirmed by Sanger sequencing using overlapping primers that covered the entire sequence.

**Table 1:** Primers used in this study.

Primers	Sequences (5′ → 3′)	Tm (°C)	Amplicon (bp)	Usage
Gap12-F	GTTGGTTGCGGTCTGTCT	53	740	Fill gaps between contigs
Gap12-R1	CATAATCACCACAAGCAAGAGA	53		
Gap12-R2	CCACAAGCAAGAGAATGAGAAG	53		
Gap23-F	GATACTGTGACTGACTGTAACG	53	650	
Gap23-R1	GCTGGAACATTCTTCAAGGATA	53		
Gap23-R2	CAGCAGGAGCAACATAATCAG	53		
5′GSP-R1	GCACTGAGGCAACGACTCGCTTACC	63	700	Terminal sequence amplification
5′GSP-R2	TGGTGCTGTTGAAGTAACTGAC	55		
3GSP-F1	GCTAGGTGGCTTGCTGTAGGTCTCATTG	63	500	
3′GSP-F2	GGCTTGCTGTAGGTCTCATTG	55		
HepQ_NS5B_fwd1	GCGTTACATCTGCTATCCTCCT	53	835	Positive sample screening
HepQ_NS5B_fwd2	AAGATGGTCCTCGGTGATGTT	53		
HepQ_NS5B_rev	CGAAGGTGAGTTGAATGGTGTT	53		

### 2.3 Sequence comparison

The identification of potential open reading frames (ORFs) was conducted using ORFfinder^1^ and annotated by comparing against the non-redundant protein database. The nucleotide sequence similarity between this newly identified *Hepacivirus* and other hepaciviruses was calculated using the MegAlign program, available in the Lasergene software package (version 7.1, DNAstar).

### 2.4 Recombination analysis

To determine potential recombination events that occurred in the evolutionary history of this newly identified *Hepacivirus*, seven methods (RDP, GENECONV, bootscan, maximum chi square, Chimera, SISCAN, and distance plot) within RDP4 program (18) were employed. Analyzes were conducted using complete genome sequences with default settings for the different test methods and a Bonferroni corrected *p*-value cutoff of 0.05. Only sequences with significant evidence (*p* < 0.05) of recombination detected by at least two methods and confirmed by phylogenetic analysis were considered to have strong evidence for recombination. Furthermore, similarity plot analyzes were performed to further characterize potential recombination events, including the location of possible breakpoints, using Simplot version 3.5.1 (19).

### 2.5 Phylogenetic analysis

To determine the phylogenetic relationship between the newly identified *Hepacivirus* and other known hepaciviruses, amino acid sequences of the complete polyprotein, NS3 (peptidase and helicase), and NS5 (RNA-dependent RNA polymerase) proteins were aligned using the E-INS-i algorithm implemented in MAFFT program (20). Phylogenetic trees were reconstructed using the maximum-likelihood method (ML) implemented in PhyML version 3.0 (21). The LG amino acid substitution model with a gamma (Γ)-distribution model (i.e., LG + Γ) determined using Prot-Test 3 (22), was employed. Bootstrap support values were calculated from 1,000 replicate trees using a Subtree Pruning and Regrafting (SPR) branch-swapping algorithm. For clarity purposes, all phylogenetic trees were rooted at the mid-point.

### 2.6 Prevalence of the newly identified *Hepacivirus*

To gain insight into the prevalence of the newly identified *Hepacivirus* circulating in domestic ducks, liver tissues were collected from live poultry markets located in five cities in Hunan (Figure 1). All individual samples, including those that subjected to meta-transcriptome sequencing, were screened for the presence of the virus using PCR with primers targeting the NS5B region (Table 1). The PCR product with the expected size was confirmed by Sanger sequencing.

### 2.7 Statistical analysis

Statistical analyzes were conducted using Statistical Package for Social Sciences (SPSS) Version 21.0 software. Descriptive statistics were used to calculate frequency and percentage, while Fisher exact test was utilized to determine the *p*-value and assess the differences in positive rates between sampling sites. A *p* value <0.05 was considered as statistically significant.

### 2.8 Ethics statement

The authors confirm that the ethical policies of the journal, as noted on the journal’s author guidelines page, have been adhered to and the appropriate ethical review committee approval has been received. The procedures for sampling and sample processing were approved by the ethics committee of Foshan University. All animals were treated in strict accordance with the Rules for the Implementation of Laboratory Animal Medicine (1998) from the Ministry of Health, China.

## 3 Results

### 3.1 Identification of a novel *Hepacivirus* in domestic ducks

**Figure 2:**
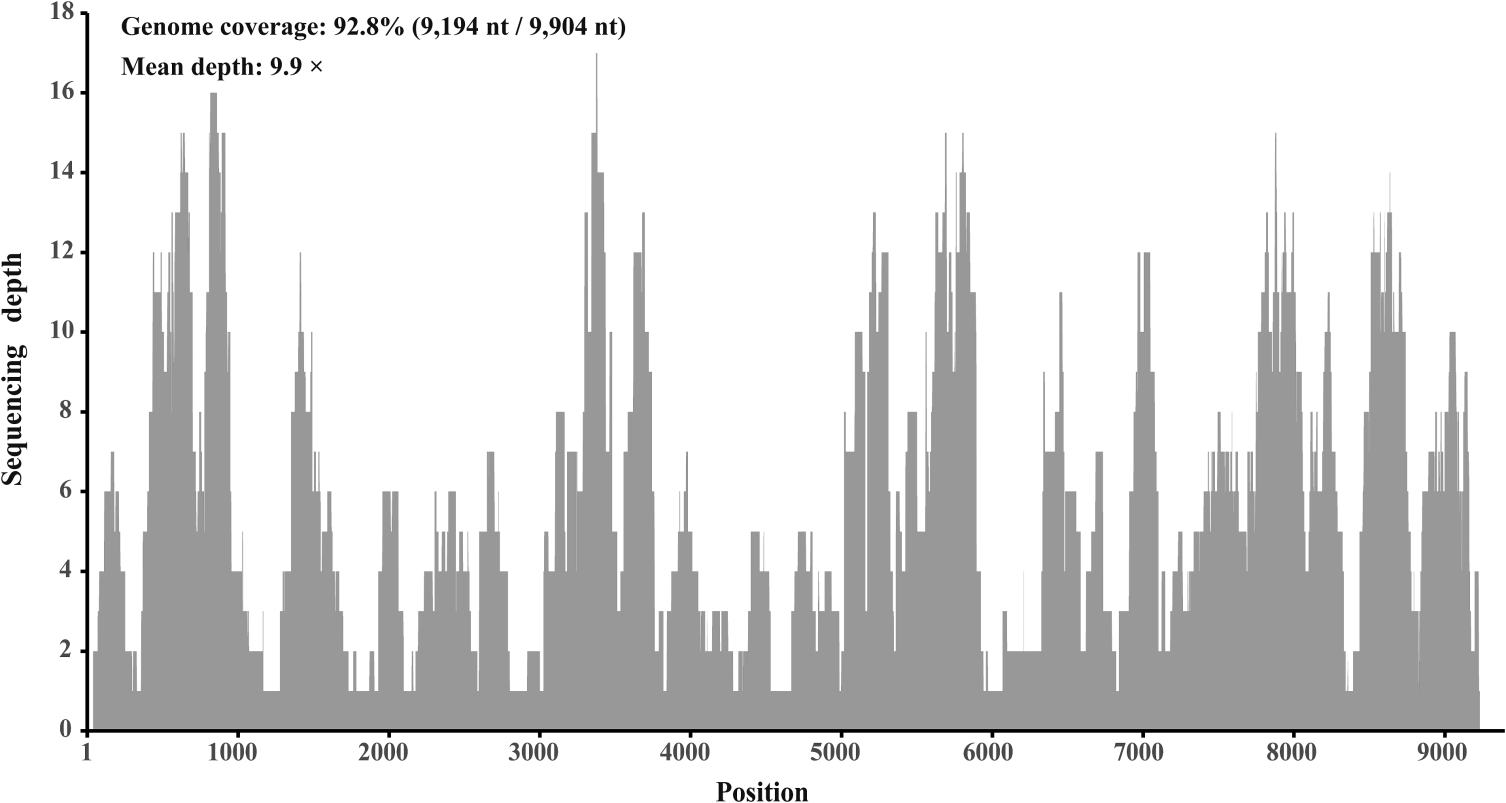
Mapped read count plot of the genome of *Hepacivirus Q* strain HN. The histograms show the coverage depth per base of genome of *Hepacivirus Q* strain HN and the mean sequencing depth of was 9.9 ×.

In a viral agent discovery project involving domestic ducks, RNA solutions extracted from 40 individual liver tissues were pooled as one sample. This sample was screened for both known and putative novel viruses through meta-transcriptome sequencing. After *de novo* assembly and comparison against the nr database, 20 contigs ranging from 257 to 995 nt in length were annotated as *Hepacivirus Q*, with 95.4 to 100% amino acid identities. After filling the gaps between these contigs through RT-PCR, and determining the terminal sequences using 5′/3′ RACE, the complete genome sequence of this virus was obtained. Using this sequence as the reference sequence, 393 reads were remapped, providing a genome coverage of 92.8% (9,194 nt/9,904 nt) and a pairwise identity of 96.2% at a mean depth of 9.9× (Figure 2).

### 3.2 Sequences comparison of this newly identified *Hepacivirus*

This newly identified *Hepacivirus* has a complete genome sequence comprising 9,893 nucleotides and contains a single large ORF that encodes a polyprotein of 2,999 amino acids. Comparative analysis of the sequence revealed that this novel virus shares a genome-wide nucleotide identity of 94.95 to 96.94% with the available sequences of *Hepacivirus Q* in GenBank. Additionally, the putative complete polyprotein of this novel *Hepacivirus* exhibits an amino acid identity of 98.13 to 98.93% with the polyprotein of *Hepacivirus Q*. Consequently, this newly identified *Hepacivirus* can be classified as a new subspecies of *Hepacivirus Q*.

### 3.3 Recombination and phylogenetic analysis

**Figure 3:**
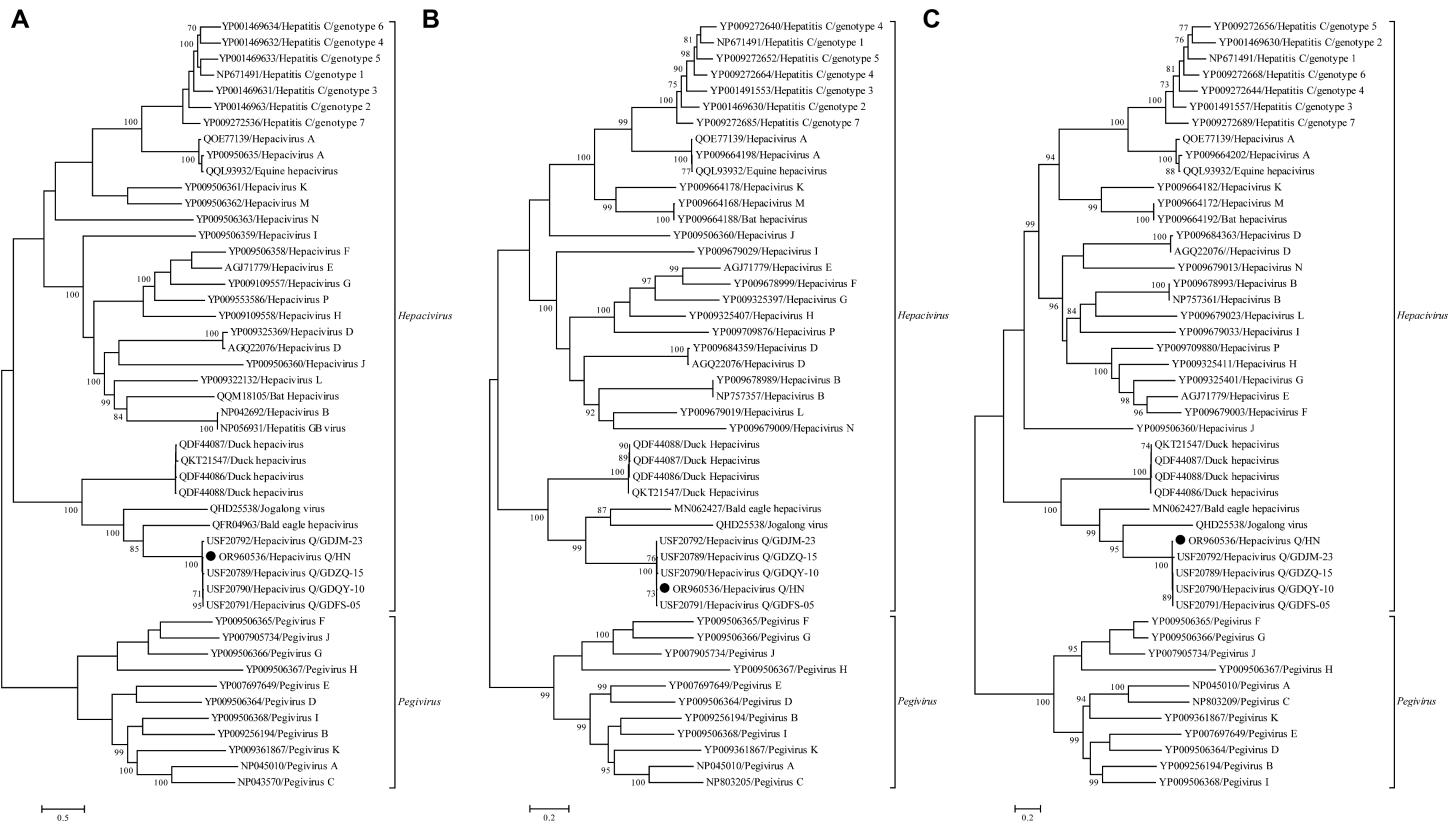
Phylogenetic analysis based on the complete polyprotein **(A)**, NS3 **(B)**, and NS5B **(C)** protein of hepaciviruses. The trees were constructed using the maximum likelihood method implemented in PhyML v3.0 and were mid-point rooted for clarity. Bootstrap values were calculated based on 100 replicates of the alignment, and only bootstrap values >70% are shown at relevant nodes. The sequence determined in this study is denoted by a black dot.

No statistically supported recombination event was detected within this novel *Hepacivirus* after systematic analyzes. Phylogenetic trees reconstructed using the amino acid sequence of the complete polyprotein, NS3, and NS5B proteins consistently revealed that this newly identified *Hepacivirus* grouped together with the known *Hepacivirus Q* strains and formed a sister lineage to a *Hepacivirus* that was identified from Bald eale in the USA (Figure 3). Moreover, the *Hepacivirus Q* group, which includes this newly identified hepacivirus, exhibits significant genetic divergence from other duck *Hepacivirus* identified in China, indicating a high level of genetic diversity within hepaciviruses. Notably, the NS3 tree shows a closer phylogenetic relationship among the *Hepacivirus Q* group and both Bald eagle *Hepacivirus* and Jogalong virus, while the NS5B tree reveals a closer relationship with Jogalong virus (Figure 3). This phylogenetic incongruence suggests that recombination events may have occurred in the evolutionary history of *Hepacivirus Q*, although no statistically supported recombination event was detected within *Hepacivirus Q* strains or other hepaciviruses during systematic analysis.

### 3.4 Prevalence of *Hepacivirus Q* in ducks in selected areas of Hunan province

A total of 480 domestic duck liver tissues, including those previously subjected to meta-transcriptomic sequencing, were screened for the presence of *Hepacivirus Q* using nested RT-PCR. Following PCR screening, sequencing, and BLAST analysis, 64 individual RNA samples tested positive for *Hepacivirus Q*, resulting in an overall prevalence rate of 13.3% (95% CI: 10.3–16.3%) (Table 2). All NS5B sequences determined herein shared 98.9 to 99.8%. Additionally, the positive rates of *Hepacivirus Q* in Hengyang, Chenzhou, Yongzhou, Shaoyang, and Huaihua were 19.2, 11.4, 10.0, 14.1, and 10.0%, respectively. No significant difference was observed among the positive rates across the different sampling sites (χ^2^ = 5.610, *p* = 0.230).

**Table 2:** Prevalence of *Hepacivirus Q* in ducks in specific regions of Hunan province.

Location	No. of individuals	No. of positive	Positive rate (%)	χ^2^-value	*p*-value
Hengyang	120	23	19.2		
Chenzhou	88	10	11.4		
Yongzhou	100	10	10.0	5.610	0.230
Shaoyang	92	13	14.1		
Huaihua	80	8	10.0		
Total	480	64	13.3		

## 4 Discussion

*Hepacivirus Q* was recently identified in domestic ducks from Guangdong province, China, utilizing meta-transcriptomic sequencing in our prior research (8). The widespread application of high-throughput sequencing has significantly aided in the unearthing of numerous new viruses, broadening our comprehension of genetic diversity and viral evolution (23). In this context, various novel hepaciviruses have been identified in a diverse array of host species (8–10, 24–30). Within this study, a novel *Hepacivirus* was uncovered in duck liver samples via meta-transcriptomic sequencing. Through sequence comparison and phylogenetic analysis, it was verified that this novel *Hepacivirus* is part of a new subspecies of *Hepacivirus Q*, highlighting the extensive genetic diversity of hepaciviruses worldwide.

In our prior investigation, *Hepacivirus Q* was observed to be present in duck populations across several cities in Guangdong province, China, exhibiting an overall prevalence of 15.9% (8). The current study identified *Hepacivirus Q* in ducks from various cities in Hunan province, China, with corresponding positive rates of 19.2, 11.4, 10.0, 14.1, and 10.0%. These results collectively suggest the broad geographical dissemination of *Hepacivirus Q* within China. Additionally, the overall positive rate of *Hepacivirus Q* in this study (13.3%) is lower than that documented in our previous study conducted in Guangdong province, China. Nevertheless, the relatively high infection rate of *Hepacivirus Q* detected in duck populations from both Guangdong and Hunan provinces highlights the necessity to investigate potential associations between *Hepacivirus Q* and duck-related ailments, although further investigations are needed for definitive.

The Hepatitis C virus, belonging to the genus *Hepacivirus*, is widely recognized as a significant causative agent of hepatitis, liver cirrhosis, and hepatocellular carcinoma in humans, presenting a substantial global public health concern (6). Despite the identification of diverse hepaciviruses in various animal species, few instances have linked animal hepaciviruses to clinical diseases (31, 32). For instance, duck *Hepacivirus* was discovered during an investigation into the etiology of severely ill ducks, with uncertain pathogenicity observed as the virus was detected in both diseased and healthy ducks (14). Similarly, *Hepacivirus Q* was identified in randomly sampled liver tissues from duck populations without any associated clinical disease manifestations in this and our previous study. However, the high prevalence of *Hepacivirus Q* in duck populations throughout southern China, across a broad geographic range, implies potential nonpathogenicity in ducks and the ability to establish persistent infections. Further study is warranted to delve into the possible pathogenicity of this novel virus in ducks.

In conclusion, a new subspecies of *Hepacivirus Q* was identified in duck specimens collected from multiple cities in Hunan province, China, exhibiting a notably high positive rate. These results enhance insights into the genetic variability and evolutionary traits of hepaciviruses, underscoring the necessity to explore potential associations between *Hepacivirus Q* and diseases affecting ducks.

## Data availability statement

The original contributions presented in the study are publicly available. This data can be found at: https://www.ncbi.nlm.nih.gov/nuccore/; OR960536.

## Ethics statement

The animal studies were approved by the ethics committee of Foshan University. The studies were conducted in accordance with the local legislation and institutional requirements. Written informed consent was not obtained from the owners for the participation of their animals in this study because all samples were collected from live poultry market.

## Author contributions

J-TC: Formal analysis, Investigation, Visualization, Writing – original draft. K-JC: Resources, Writing – original draft. K-WW: Resources, Writing – original draft. S-HY: Investigation, Writing – original draft. J-WS: Conceptualization, Funding acquisition, Writing – review & editing.

## Footnotes

^1^ https://www.ncbi.nlm.nih.gov/orffinder

## References

[1] Smith DB Becher P Bukh J Gould EA Meyers G Monath T. Proposed update to the taxonomy of the genera Hepacivirus and Pegivirus within the Flaviviridae family. J Gen Virol. (2016) 97:2894–907. doi: 10.1099/jgv.0.000612, PMID: 27692039 PMC5770844

[2] Simmonds P Becher P Bukh J Gould EA Meyers G Monath T. ICTV virus taxonomy profile: Flaviviridae. J Gen Virol. (2017) 98:2–3. doi: 10.1099/jgv.0.000672, PMID: 28218572 PMC5370391

[3] Scheel TK Simmonds P Kapoor A. Surveying the global virome: identification and characterization of HCV-related animal hepaciviruses. Antivir Res. (2015) 115:83–93. doi: 10.1016/j.antiviral.2014.12.014, PMID: 25545071 PMC5081135

[4] Penin F Dubuisson J Rey FA Moradpour D Pawlotsky JM. Structural biology of hepatitis C virus. Hepatology. (2004) 39:5–19. doi: 10.1002/hep.20032 14752815

[5] Boonstra A van der Laan LJ Vanwolleghem T Janssen HL. Experimental models for hepatitis C viral infection. Hepatology. (2009) 50:1646–55. doi: 10.1002/hep.23138 19670425

[6] Hartlage AS Cullen JM Kapoor A. The strange, expanding world of animal hepaciviruses. Annu Rev Virol. (2016) 3:53–75. doi: 10.1146/annurev-virology-100114-055104, PMID: 27741408 PMC5523456

[7] Li LL Liu MM Shen S Zhang YJ Xu YL Deng HY. Detection and characterization of a novel hepacivirus in long-tailed ground squirrels (*Spermophilus undulatus*) in China. Arch Virol. (2019) 164:2401–10. doi: 10.1007/s00705-019-04303-z, PMID: 31243554

[8] Zhang XL Yao XY Zhang YQ Lv ZH Liu H Sun J. A highly divergent hepacivirus identified in domestic ducks further reveals the genetic diversity of hepaciviruses. Viruses. (2022) 14:371. doi: 10.3390/v14020371 35215964 PMC8879383

[9] Chu L Jin M Feng C Wang X Zhang D. A highly divergent hepacivirus-like flavivirus in domestic ducks. J Gen Virol. (2019) 100:1234–40. doi: 10.1099/jgv.0.001298, PMID: 31282853

[10] Lu G Zhao J Ou J Li S. Novel HCV-like virus detected in avian livers in southern China and its implications for natural recombination events. Virol Sin. (2021) 36:149–51. doi: 10.1007/s12250-020-00256-9, PMID: 32617899 PMC7973315

[11] Goldberg TL Sibley SD Pinkerton ME Dunn CD Long LJ White LC. Multidecade mortality and a homolog of hepatitis C virus in bald eagles (*Haliaeetus leucocephalus*), the national bird of the USA. Sci Rep. (2019) 9:14953. doi: 10.1038/s41598-019-50580-8, PMID: 31628350 PMC6802099

[12] Shi M Lin XD Chen X Tian JH Chen LJ Li K. The evolutionary history of vertebrate RNA viruses. Nature. (2018) 556:197–202. doi: 10.1038/s41586-018-0012-7 29618816

[13] Zhang XL Li WF Yuan S Guo JY Li ZL Chi SH. Meta-transcriptomic analysis reveals a new subtype of genotype 3 avian hepatitis E virus in chicken flocks with high mortality in Guangdong, China. BMC Vet Res. (2019) 15:131. doi: 10.1186/s12917-019-1884-y 31060564 PMC6503432

[14] Chen S Zhou Y Chen Y Gu J. Fastp: an ultra-fast all-in-one FASTQ preprocessor. Bioinformatics. (2018) 34:i884–90. doi: 10.1093/bioinformatics/bty560, PMID: 30423086 PMC6129281

[15] Li D Liu CM Luo R Sadakane K Lam TW. MEGAHIT: an ultra-fast single-node solution for large and complex metagenomics assembly via succinct de Bruijn graph. Bioinformatics. (2015) 31:1674–6. doi: 10.1093/bioinformatics/btv033, PMID: 25609793

[16] Buchfink B Xie C Huson DH. Fast and sensitive protein alignment using DIAMOND. Nat Methods. (2015) 12:59–60. doi: 10.1038/nmeth.3176, PMID: 25402007

[17] Langmead B Salzberg SL. Fast gapped-read alignment with bowtie 2. Nat Methods. (2012) 9:357–9. doi: 10.1038/nmeth.1923, PMID: 22388286 PMC3322381

[18] Martin DP Murrell B Golden M Khoosal A Muhire B. RDP4: detection and analysis of recombination patterns in virus genomes. Virus Evol. (2015) 1:vev003. doi: 10.1093/ve/vev003 27774277 PMC5014473

[19] Lole KS Bollinger RC Paranjape RS Gadkari D Kulkarni SS Novak NG. Full-length human immunodeficiency virus type 1 genomes from subtype C-infected seroconverters in India, with evidence of intersubtype recombination. J Virol. (1999) 73:152–60. doi: 10.1128/JVI.73.1.152-160.1999, PMID: 9847317 PMC103818

[20] Katoh K Standley DM. MAFFT multiple sequence alignment software version 7: improvements in performance and usability. Mol Biol Evol. (2013) 30:772–80. doi: 10.1093/molbev/mst010, PMID: 23329690 PMC3603318

[21] Guindon S Gascuel O. A simple, fast, and accurate algorithm to estimate large phylogenies by maximum likelihood. Syst Biol. (2003) 52:696–704. doi: 10.1080/10635150390235520, PMID: 14530136

[22] Darriba D Taboada GL Doallo R Posada D. ProtTest 3: fast selection of best-fit models of protein evolution. Bioinformatics. (2011) 27:1164–5. doi: 10.1093/bioinformatics/btr088, PMID: 21335321 PMC5215816

[23] Zhang YZ Chen YM Wang W Qin XC Holmes EC. Expanding the RNA virosphere by unbiased metagenomics. Annu Rev Virol. (2019) 6:119–39. doi: 10.1146/annurev-virology-092818-015851, PMID: 31100994

[24] Lu G Jia K Ping X Huang J Luo A Wu P. Novel bovine hepacivirus in dairy cattle, China. Emerg. Microbes Infect. (2018) 7:1–3. doi: 10.1038/s41426-018-0055-8 PMC5883034 29615608

[25] Canal CW Weber MN Cibulski SP Silva MS Puhl DE Stalder H. A novel genetic group of bovine hepacivirus in archival serum samples from Brazilian cattle. Biomed Res Int. (2017) 2017:1–4. doi: 10.1155/2017/4732520 PMC5585675 28904959

[26] Porter AF Pettersson JH Chang WS Harvey E Rose K Shi M. Novel hepaci-and pegi-like viruses in native Australian wildlife and non-human primates. Virus Evol. (2020) 6:veaa064. doi: 10.1093/ve/veaa064 33240526 PMC7673076

[27] Guo H Cai C Wang B Zhuo F Jiang R Wang N. Novel hepacivirus in Asian house shrew, China. Sci China Life Sci. (2019) 62:701–4. doi: 10.1007/s11427-018-9435-7, PMID: 30701456 PMC7088713

[28] de Souza WM Fumagalli MJ Sabino-Santos G Jr Motta Maia FG Modha S Teixeira Nunes MR. A novel hepacivirus in wild rodents from South America. Viruses. (2019) 11:297. doi: 10.3390/v11030297 30909631 PMC6466192

[29] Shao JW Guo LY Yuan YX Ma J Chen JM Liu Q. A novel subtype of bovine hepacivirus identified in ticks reveals the genetic diversity and evolution of bovine hepacivirus. Viruses. (2021) 13:2206. doi: 10.3390/v13112206 34835012 PMC8623979

[30] Breitfeld J Fischer N Tsachev I Marutsov P Baymakova M Plhal R. Expanded diversity and host range of bovine hepacivirus-genomic and serological evidence in domestic and wild ruminant species. Viruses. (2022) 14:1457. doi: 10.3390/v14071457 35891438 PMC9319978

[31] Baechlein C Fischer N Grundhoff A Alawi M Indenbirken D Postel A. Identification of a novel hepacivirus in domestic cattle from Germany. J Virol. (2015) 89:7007–15. doi: 10.1128/JVI.00534-15, PMID: 25926652 PMC4473572

[32] Reuter G Maza N Pankovics P Boros A. Non-primate hepacivirus infection with apparent hepatitis in a horse—short communication. Acta Vet Hung. (2014) 62:422–7. doi: 10.1556/avet.2014.011 25038950

